# Spectral Regression Based Fault Feature Extraction for Bearing Accelerometer Sensor Signals

**DOI:** 10.3390/s121013694

**Published:** 2012-10-12

**Authors:** Zhanguo Xia, Shixiong Xia, Ling Wan, Shiyu Cai

**Affiliations:** School of Computer Science and Technology, China University of Mining and Technology, Xuzhou 221116, Jiangsu, China; E-Mails: wanling@cumt.edu.cn (L.W.); caishy@tom.com (S.C.)

**Keywords:** feature extraction, spectral regression, bearing accelerometer sensor, fault diagnosis, fault prognosis

## Abstract

Bearings are not only the most important element but also a common source of failures in rotary machinery. Bearing fault prognosis technology has been receiving more and more attention recently, in particular because it plays an increasingly important role in avoiding the occurrence of accidents. Therein, fault feature extraction (FFE) of bearing accelerometer sensor signals is essential to highlight representative features of bearing conditions for machinery fault diagnosis and prognosis. This paper proposes a spectral regression (SR)-based approach for fault feature extraction from original features including time, frequency and time-frequency domain features of bearing accelerometer sensor signals. SR is a novel regression framework for efficient regularized subspace learning and feature extraction technology, and it uses the least squares method to obtain the best projection direction, rather than computing the density matrix of features, so it also has the advantage in dimensionality reduction. The effectiveness of the SR-based method is validated experimentally by applying the acquired vibration signals data to bearings. The experimental results indicate that SR can reduce the computation cost and preserve more structure information about different bearing faults and severities, and it is demonstrated that the proposed feature extraction scheme has an advantage over other similar approaches.

## 1. Introduction

Bearings are one of the most important components in rotating machinery [1]. Many of the faults of rotating machinery relate to the bearings, whose running conditions directly affect the precision, reliability and life of the machine [2]. Breakdowns caused by bearing performance degradation and inappropriate operation can not only lead to huge economic losses for enterprises, but also potentially serious casualties [3]. In recent years, therefore, bearings fault prognosis technology has received more and more attention, in particularly fault feature extraction (FFE) of bearing accelerometer sensor signals has become more and more important in order to avoid the occurrence of accidents.

Bearing accelerometer sensor signal analysis-based techniques, which are the most suitable and effective ones for bearing, have been extensively used since in machine prognosis it is easy to obtain sensor signals containing abundant information. These techniques mainly include three categories, namely, time domain analysis, frequency domain analysis and time-frequency domain analysis. Time domain analysis calculates characteristic features of signals statistics such as root mean squares (RMS), kurtosis value, skewness value, peak-peak value, crest factor, impulse factor, margin factor, *etc.* Frequency domain analysis search for a train of ringing occurring at any of the characteristic defect frequencies, it is widely applied in fast Fourier transform (FFT), spectrum analysis, envelop analysis, *etc.* Time-frequency domain analysis investigates given signals in both the time and frequency domain, which is successfully developed for non-stationary signals, and some different technologies such as short-time Fourier transform (STFT), wavelet transform (WT), wavelet packet transform (WPT), Hilbert-Huang transform (HHT), *etc.* are described in the literature [3–6]. Among them, energy features of reconstructed vibration signals are commonly calculated for the purpose of signal analysis, for example, the wavelet energy can represent the characteristics of vibration signals. Consequently, a lot of original features can be generated from accelerometer sensor signals, therefore it is a necessity to deal with large scale feature dimensions. The biggest challenge is how to extract the most useful information that can reflect comprehensive performance degradation. Previous research has shown that different features are sensitive to different faults and degradation stages, for example, kurtosis value and crest factor are sensitive to impulse faults, especially in the incipient stage, but they will decrease to normal-like levels as the damage grows, which shows that the stability of these features is not satisfactory [7].

Feature extraction means transforming the existing features into a lower dimensional space which is useful for feature reduction to avoid the redundancy due to high-dimensional data [8]. Principal component analysis (PCA) might be one of feature extraction techniques which is often used for bearing fault detection or classification, PCA has the ability to discriminate directions with the largest variance in a data set, and extract several representative features by using data projection. Factor analysis (FA) is a statistical method used to describe variability among observed, correlated variables in terms of a potentially lower number of unobserved variables called factors, FA has been demonstrated to be able to extract important knowledge from sensor data based on the inter-correlation of sensor data [9]. Locality preserving projections (LPP) is a linear projective map that arises by solving a variational problem that optimally preserves the intrinsic geometry structure of the dataset in a low-dimensional space. Liao and Lee in [10] used PCA to find the first two principal components (wavelet packet node energy) which contain more than 90 percent of the variation information. Widodo and Yang in [11] employed PCA to obtain one dimensional features of condition monitor histories from which the survival probability of the historical event data. Côme and Oukhellou in [12] applied the independent factor analysis for intelligent fault diagnosis of railway track circuits, and the diagnosis system aimed to recover the latent variables linked to track circuit defects using features extracted, significantly improving estimation accuracy and removing indeterminacy. Yu in [13] used LPP to extract the most representative features for representing the bearing performance, indicating that LPP could find more meaningful low-dimensional information hidden in the high dimensional feature set compared with PCA. PCA, FA and LPP play a manifest role in feature extraction, however, they have their limitations and don't contain a full exploitation of the multivariate nature of the data [14].

Spectral methods have recently emerged as a powerful tool for dimensionality reduction and manifold learning [15], these methods use information contained in the eigenvectors of a data affinity matrix to reveal low dimensional structure in high dimensional data. Spectral regression (SR) is a novel regression framework for efficient regularized subspace learning and feature extraction technology [16]. Different from other similar methods, SR combines the spectral graph analysis and regression to provide an efficient and effective approach for regularized subspace learning problem. It is shown that SR casts the problem of learning an embedding function into a regression framework, which avoids eigen-decomposition of dense matrices. Due to its superior properties, for example, the lower computation cost and the more structured information, it can be used in all unsupervised, semi-supervised or supervised problems. SR has been adopted for various applications such as location of the sensor nodes [17], human action recognition [18], facial image retrieval [19], EEG signals [20], *etc.* To the best of our knowledge, no research results have been published to data on the use of SR for bearing fault feature extraction and machine prognosis, therefore, this paper will be the first time SR was applied to feature extraction of bearing faults.

The rest of the paper is organized as follows: Section 2 proposes the signal processing (including feature calculation) from accelerometer sensors according to the time domain, frequency domain and time-frequency domain. In Section 3, the graph embedding view and SR-based feature extraction approach are introduced. Section 4 gives a description of the experiments and analysis, bearing accelerometer sensor signals from bearings are employed to evaluate the effectiveness of the proposed method. Finally, concluding remarks and future work on this approach are given in Section 5.

## 2. Signal Processing from Accelerometer Sensor

To diagnose the abnormality, it is important to record certain physical parameters which vary according to the variation in the operation of the machine [21]. Vibration signals are extensively used in signature matching for abnormality detection and diagnosis. Generally, these signals are generated by accelerometer sensors on bearings [22]. The essential aim of signal processing is to map a signal from the time domain into another space in which some important information of the signals can be revealed, and consequently, some dominant features of the signals can be extracted [23]. For this purpose, various original features that can be extracted from accelerometer sensor signals of bearings have been investigated. This section presents a brief discussion of feature generation from time-domain, frequency-domain, and time-frequency domain as they will be used throughout the paper.

Time domain features often involve statistical features that are sensitive to impulse faults [13], especially in the incipient stage, so we calculated some dimensional features, such as RMS, square root of the amplitude (SRA), kurtosis value (KV), skewness value (SV) and peak-peak value (PPV), in addition, some dimensionless features, such as crest factor (CF), impulse factor (IF), margin factor (MF), shape factor (SF) and kurtosis factor (KF). These features are defined as follows:

(1) $$X_{\text{rms}}={\left(\frac{1}{N}\sum_{i=1}^{N}x_{i}^{2}\right)}^{1/2}$$

(2) $$X_{\text{sra}}={\left(\frac{1}{N}\sum_{i=1}^{N}\sqrt{\left|x_{i}\right|}\right)}^{2}$$

(3) $$X_{\text{kv}}=\frac{1}{N}\sum_{i=1}^{N}{\left(\frac{x_{i}-\bar{x}}{\sigma }\right)}^{4}$$

(4) $$X_{\text{sv}}=\frac{1}{N}\sum_{i=1}^{N}{\left(\frac{x_{i}-\bar{x}}{\sigma }\right)}^{3}$$

(5) $$X_{\text{ppv}}=\max(x_{i})-\min(x_{i})$$

(6) $$X_{\text{cf}}=\max(\left|x_{i}\right|)/{\left(\frac{1}{N}\sum_{i=1}^{N}x_{i}^{2}\right)}^{1/2}$$

(7) $$X_{\text{if}}=\max(\left|x_{i}\right|)/\frac{1}{N}\sum_{i=1}^{N}\left|x_{i}\right|$$

(8) $$X_{\text{mf}}=\max(\left|x_{i}\right|)/{\left(\frac{1}{N}\sum_{i=1}^{N}\sqrt{\left|x_{i}\right|}\right)}^{2}$$

(9) $$X_{\text{sf}}={\left(\frac{1}{N}\sum_{i=1}^{N}x_{i}^{2}\right)}^{1/2}/\frac{1}{N}\sum_{i=1}^{N}\left|x_{i}\right|$$

(10) $$X_{\text{kf}}=\frac{1}{N}\sum_{i=1}^{N}{\left(\frac{x_{i}-\bar{x}}{\sigma }\right)}^{4}/{\left(\frac{1}{N}\sum_{i=1}^{N}x_{i}^{2}\right)}^{2}$$

Frequency domain analysis is another description of a signal, that can reveal some information that cannot be found in the time domain [24]. Frequency domain features are calculated on the basis of FFT from time domain vibration signals, these features often involve statistical results of frequency, such as frequency center (FC), RMS frequency (RMSF) and root variance frequency (RVF), *etc.* These features are defined as follows:

(11) $$X_{\text{fc}}=\int_{0}^{+\infty }fs(f)df/\int_{0}^{+\infty }s(f)df$$

(12) $$X_{\text{rmsf}}={\left(\int_{0}^{+\infty }f^{2}s(f)df/\int_{0}^{+\infty }s(f)df\right)}^{1/2}$$

(13) $$X_{\text{rvf}}={\left(\int_{0}^{+\infty }{\left(f-X_{fc}\right)}^{2}s(f)df/\int_{0}^{+\infty }s(f)df\right)}^{1/2}$$

**Figure 1.**
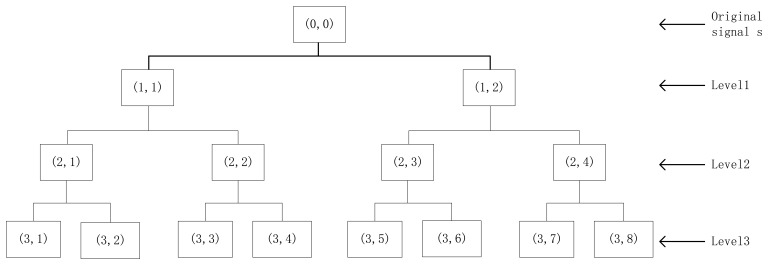
An example of three-level wavelet packet decomposition.

Time-frequency domain methods are considered to be best way for analyzing non-stationary signals [25], due to the deficiency of the Fourier transform. Many time-frequency analysis technologies have been developed, including STFT, WT (or WPT), HHT, *etc.* In this study, we adopt WPT to present bearing accelerometer sensor signals in time-frequency distribution diagrams with multi-resolution. As we know, wavelet packet analysis (WPA) is an extension of WT which provides complete level-by-level decomposition. As shown in Figure 1, wavelet packets are particular linear combinations wavelets. The wavelet packets inherit properties such as orthogonality, smoothness and time-frequency localization from their corresponding wavelet functions. Let *Ψ* be a wavelet packet function with three integer indices *i*, *j* and *k* which are the modulation or oscillation parameter, the scale parameter, and the translation parameter, respectively:

(14) $$\psi =\psi_{j,k}^{i}(t)=2^{j/2}\psi^{i}(2^{j}t-k)$$

The wavelet packet coefficients of a signal *s* can be computed by taking the inner product of the signal and the wavelet packet function:

(15) $$c_{j,k}^{i}=\langle s,\psi_{j,k}^{i}(t)\rangle =\int_{-\infty }^{+\infty }s(t)\psi_{j,k}^{i}(t)dt$$

The wavelet packet node energy *WPNE*(*j,k*) can represent the characteristics of vibration signals, and it is defined as:

(16) $$\text{WPNE}(j,k)=\sum_{k}{\left(c_{j,k}^{i}\right)}^{2}$$

In this application, we use a specific wavelet function “DB4” from the Daubechies (DB) wavelets family as the mother wavelet and decompose the vibration signals into four levels. In general, the biggest challenge in wavelet analysis is the selection and determination of the mother wavelet function as well as the decomposition level of signals for the different real-world applications [21]. Different mother wavelet functions and corresponding orders have different effects on the feature extraction. Rafiee *etc.* in [26] presented a novel solution to find the best mother wavelet function for fault classification purposes as well as the best level of decomposing the vibration signals by wavelet analysis in machine condition monitoring; the experimental results demonstrated that a DB4 orthogonal wavelet discloses abnormal transients generated by the bearing damage from the vibration signals more effectively than other wavelets in the range of DB2 and DB20, and the optimized value of the decomposition level is 4. In addition, a large number of previous studies have demonstrated that DB4 has been widely implemented as it matches the transient components in vibration signals and showed effectiveness in defect detection and fault diagnosis of bearings, because it has the advantages of orthogonality and computational simplicity [27]. Subsequently, we calculate wavelet packet node energy in fourth level as the input features of bearing time-frequency domain:

(17) $$X_{\text{wpne}}(j,k)=\text{WPNE}(j,k)$$

## 3. SR-Based Feature Extraction Approach

In this section, after the graph embedding and SR method are presented, SR-based fault feature extraction approach is proposed to extract useful information from the calculated original features of vibration signals.

### 3.1. Graph Embedding

The SR is fundamentally based on regression theory and spectral graph analysis, so it can be incorporated into other algorithms easily [28]. It can be used in all unsupervised, semi-supervised or supervised problems and integrated with different other suggested regularizers to make it more flexible [29]. In concrete applications, an affinity graph will be constructed first via the labeled and unlabeled samples, in order to reveal the intrinsic structured information and to learn the responses with the given data. Subsequently, with these obtained responses, the ordinary regression is applied for learning the embedding function.

The SR aims at finding a low-dimensional subspace *Z* = [**z**_1_, **z**_2_, …, **z***_m_*] (**z***_i_*∈*R^d^*), when given high-dimensional input data *X* = [**x**_1_, **x**_2_, …, **x***_m_*] (**x***_i_*∈*R^n^*, *d* ≪ *n*), where *m* is the sample number, say **x***_i_* can be represented with **z***_i_*. Let **x** = [*x*_1_, *x*_2_, …, *x_m_*]*^T^* be high-dimensional space and y = [*y*_1_, *y*_2_, …, *y_m_*]*^T^* be the mapped low-dimensional space, a reasonable criterion for choosing a map is to minimize:

(18) $$\sum_{i,j}{\left\|y_{i}-y_{j}\right\|}^{2}W_{ij}$$

where the matrix *W_ij_* with *m* × *m* entries contains the weight of the edge, these edges join points *x_i_* and *x_j_* in a nearest-neighbor graph *G* with *m* points. The objective function will be heavily penalized if neighboring points *x_i_* and *x_j_* are mapped far apart. Therefore, the purpose of minimizing is to ensure that if *x_i_* and *x_j_* are “close” then *y_i_* and *y_j_* are close as well. Following some algebraic steps, we have:

(19) $$\frac{1}{2}\sum_{i,j}{\left\|y_{i}-y_{j}\right\|}^{2}W_{ij}=y^{T}(D-W)y=y^{T}Ly$$

where *D* is a diagonal matrix, which contains column sums of *W*, *D_ii_* = Σ*_j_W_ji_*, and *L* = *D* − *W* is the graph Laplacian matrix. And then, the minimization problem in Equation (18) reduces to finding:

(20) $$y^{*}=\underset{y^{T}Dy=1}{\arg\min}y^{T}Ly=\text{argmin}\frac{y^{T}Ly}{y^{T}Dy}$$

In order to remove the arbitrary scaling factor in the embedding, a constraint **y**^T^*D***y** = 1 will be imposed. Obviously, it is because of *L* = *D* − *W*, Equation (20) is also equivalent to the maximization problem:

(21) $$y^{*}=\underset{y^{T}Dy=1}{\arg\max}y^{T}Wy=\text{argmax}\frac{y^{T}Wy}{y^{T}Dy}$$

The optimal *y*'s in Equation (21) can be obtained by solving the generalized eigenvalue problem:

(22) Wy=λDy

For simply mapping for training samples and new testing samples, we choose a linear function here:

(23) $$\begin{array}{cc} y_{i}=f(x_{i})=A^{T}x_{i}, & A^{T}=(a_{1},\cdots \end{array}$$

where ***A*** is a *n*×*d* matrix, *x_i_* is mapped to *y_i_*. Substituting Equation (23) into Equation (21), we have:

(24) $$A^{*}=\text{argmax}\frac{y^{T}Wy}{y^{T}Dy}=\text{argmax}\frac{A^{T}XWX^{T}A}{A^{T}XDX^{T}A}$$

The optimal ***A***'s in Equation (24) can be also obtained by solving the generalized eigenvalue problem:

(25) $${\text{XWX}}^{T}A=\lambda {\text{XDX}}^{T}A$$

This maximum eigen-problem formulation in some cases can provide a more numerically stable solution. In the remainder of this paper, we will develop the SR algorithm based on Equation (25).

### 3.2. Spectral Regression Algorithm

The SR has been used in various applications where it has demonstrated efficacy compared to PCA, FA, and some common manifold techniques in both feature quality and calculation efficiency [15]. Meanwhile, the SR algorithm uses the least squares method to get the best projection direction, rather than computing the density matrix of features, so it also has an advantage in speed. An affinity graph *G* of both labeled and unlabeled points is constructed to find the intrinsic discriminant structure and to learn the responses with the given data. Then, with these responses, the ordinary regression is applied for learning the embedding function [30].

Given a training set with *l* labeled samples **x**_1_, **x**_2_, …, **x***_l_* and a testing set with (*m* − *l*) unlabeled samples **x***_l_*_+1_, **x***_l_*_+2_, …, **x***_m_*, where the sample **x***_i_*∈*R^n^* belongs to one of *c* classes, and let *l_k_* be the number of labeled samples in the *k*-th class (the sum of *l_k_* is equal to *l*). The SR is summarized as follows:

**Step1:** Constructing the adjacency graph *G*: Let **X** be the training set and *G* denote a graph with *m* nodes, where the *i*-th node corresponds to the sample **x***_i_*. In order to model the local structure as well as the label information, then the graph *G* will be constructed through the following three steps:
  1. If **x***_i_* is among *p* nearest neighbors of **x***_j_* or **x***_j_* is among *p* nearest neighbors of **x***_i_*, then nodes *i* and *j* are connected by an edge;
  2. If **x***_i_* and **x***_j_* are in the same class (*i.e.*, same label), then nodes *i* and *j* are also connected by an edge;
  3. Otherwise, if **x***_i_* and **x***_j_* are not in the same class, then the edge will be deleted between nodes *i* and *j.*
**Step2:** Constructing the weight matrix *W*: Let *W* b*e* the sparse symmetric *m*×*m* matrix, where *W_ij_* having the weight of the edge joining vertices *i* and *j*.
  1. If there is no any edge between nodes *i* and *j*, then *W_ij_* = 0;
  2. Otherwise, if both **x***_i_* and **x***_j_* belong to the *k*-th class, then *W_ij_* = 1/*l_k_*, *else W_ij_* =_δ_. *s*(*i*, *j*), where _δ (_0 < _δ_ ≤ 1) is a given parameter to adjust the weight between supervised and unsupervised neighbor information. Therein, *s*(*i*, *j*) is a similarity evaluation function between **x***_i_* and **x***_j_*, there are three variations, the first one is Simple-minded function *s*(*i*, *j*) = 1, the second one is Heat kernel function:
    (26) $$s(i,j)=\exp\left(-{\left\|x_{i}-x_{j}\right\|}^{2}/2\sigma^{2}\right)$$
    where *σ*∈*R*, the third one is Cosine weight:
    (27) $$s(i,j)=x_{i}^{T}x_{j}/\left\|x_{i}\right\|\left\|x_{i}\right\|$$
**Step3:** Eigen-decomposing: Let *D* be the *m* × *m* diagonal matrix, whose the (*i*, *i*)-th element is the sum of the *i*-th column (or row) of *W*. Find *y*_0_, *y*_1_, …, *y_c_*_−1_, which are the largest *c* generalized eigenvectors of eigen-problem:
  (28) Wy=λDy
  where the first eigenvector *y*_0_ is a vector of all ones with eigenvalue 1.
Step4: Regularized least squares: Calculate *c*-1vectors *a*_1_, …, *a_c_*_−1_ with *a_k_*∈*R^n^* (*k* = 1, …, *c*−1), therein *a_k_* is then a solution of regularized least square problem:
  (29) $$a_{k}=\underset{a}{\arg\min}\left(\sum_{i=1}^{m}{\left(a^{T}x_{i}-y_{i}^{k}\right)}^{2}+\alpha {\left\|a\right\|}^{2}\right)$$
  where $y_{i}^{k}$ is the *i*-th element of *y_k_*. In order to obtain *a_k_*, the following linear equations system can be used to solve through the classic Gaussian elimination method.
  (30) $$\left(XX^{T}+\alpha I\right)a_{k}=Xy_{k}$$
  where *I* is a *n* × *n* identity matrix.
**Step5:** SR Embedding: Let *A* be an *n* × (*c* − 1) obtained transformation matrix through the previously mentioned processes, where *A* = [*a*_1_, …, *a_c_*_−1_]. The testing samples or new sample can be embedded into *c* − 1 dimensional subspace by:
  (31) $$x\to z=A^{T}x$$

### 3.3. SR-Based Fault Feature Extraction

**Figure 2.**
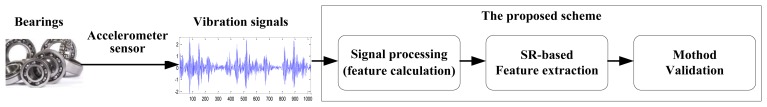
The flow chart of the proposed scheme.

Feature extraction, which is a mapping process from the measured signal space to the feature space, can be regarded as the most important step for intelligent fault diagnosis systems [14]. The effective feature extraction is important for the pattern recognition of bearing faults [31]. In this work, we propose an SR-based fault feature extraction scheme for bearing accelerometer sensor signals, The flow chart of the proposed scheme is shown in Figure 2, which includes three parts: *i.e.*, signal processing (or named as feature calculation), feature extraction and method evaluation.

Firstly, we calculate 10 features of the time domain directly from bearing vibration signals and three features of the frequency domain based on FFT. Subsequently, we decompose vibration signals into four scales using WPT with ‘DB4’, and then calculate wavelet packet nodes energy in the fourth level as 16 features of the time-frequency domain. So far, we have obtained 29 initial features from vibration signals (see Table 1), which have been enough to represent the bearing performance states and fault severity. As we know, because it is difficult to estimate which features are more sensitive to defect development and propagation in a machine system, as various factors affect the effectiveness of the features. In this case, we believe that it is more helpful to generate more and more various features.

Secondly, we extract the most representative features from 29 initial features via the SR-based method. Obviously, very large initial features' dimension will result in decreasing performance of bearing prognosis and therefore also increasing computational costs. How to extract the really effective information of bearing fault is a challenging problem. In this paper, if we choose the first *d* eigenvectors from *A* = [*a*_1_, …, *a_c_*_−1_] in Equation (31), where *d* ≫ *c* − 1, then the new projection z is:

(32) $$z=A^{T}x,A=(a_{1},\ldots ,a_{d}).$$

Based on the new projection data set z using SR-based method, the high-dimensional data space is reduced to a low-dimensional data space, however, retaining the majority of local variation information in the projected data set. With the reduced dimensions and local variance information preservation, the extracted features z will be used as the new input features of pattern recognizers for bearing faults.

Finally, we validate the SR-based method using *K*-means in the case of original features and extracted features. In this paper, we compare the SR-based method with PCA-based, FA-based ones, *etc.*, and the experiment results show that SR-based method is the best for extracting the useful information to represent bearing performance conditions from the available original features as you can calculate from the vibration signals. Moreover, the validated result confirms that the features extracted by SR ensure effective fault recognition at higher accuracy than the 29 original features.

## 4. Experiments and Analysis

### 4.1. Data Acquisition

Data acquisition is a process of collecting and storing useful data from targeted physical assets for the purpose of Condition-based Maintenance (CBM). This process is an essential step in implementing a CBM program for machinery fault diagnosis and prognosis. To evaluate the effectiveness of the signal processing and feature extraction methods for bearings, the vibration data related to the bearing and the system investigation in this paper were provided by the Bearing Data Center of the Case Western Reserve University (CWRU), and acquired by bearing accelerometer sensors under different operating loads and bearing conditions [32]. The bearing data of CWRU has been validated in many research works and become a standard dataset for bearing studies [2,13,14,21].

**Figure 3.**
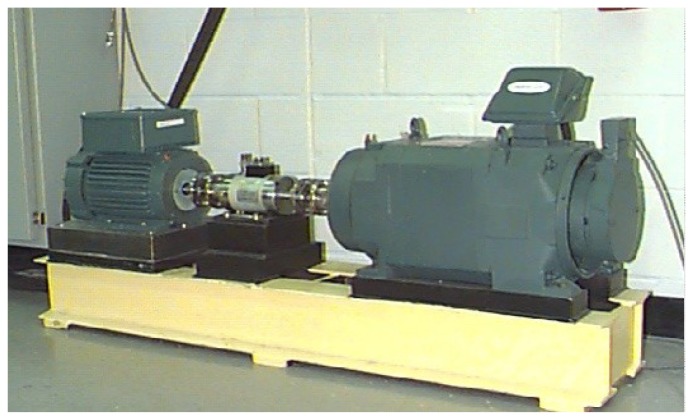
The test-rig.

The test-rig shown in Figure 3 consists of a 2 HP motor (left), a torque transducer/encoder (center), a dynamometer (right), and control electronics (not shown). The test bearing type is a 6205-2RS JEM SKF, which is a deep groove ball bearing, the dynamometer is controlled so that desired torque load levels can be achieved. Accelerometer sensors were placed at the 12 o'clock position at the drive end of the motor housing. The experimental rotating frequency is about 30 Hz, the test bearings support the motor shaft and the load was 2 HP at the speed of 1,797 rpm, single point faults were introduced to the inner race, ball and outer race of the test bearings using electro-discharge machining (EDM) with fault diameters of 0.007, 0.014, 0.021 and 0.028 inches, and the fault depth is 0.011 inches. More detailed information about the test-rig can be found in [32].

The vibration signals were collected through accelerometers using a 16 channel digital audio tape (DAT) recorder at the sampling frequency 12 kHz. In order to evaluate the performance of the SR-based feature extraction approach proposed in this paper, we separate the experimental vibration data into four datasets, named as D_IRF, D_ORF, D_BF and D_MIX. Specifically, similar to the ORF and BF datasets, the IRF dataset includes five severity conditions, *i.e.*, normal, and four types of fault bearings with faulty diameter: 0.007 (IRF07), 0.014 (IRF14), 0.021 (IRF21) and 0.028 (IRF28) inches in the inner race of the bearings, respectively. The D_MIX dataset, however, contains four different states which are normal, and three types of faults, *i.e.*, inner race fault (IRF), ball fault (BF) and outer race fault (ORF) all with a fault diameter of 0.014 inches. The length of the signal data in every dataset is 1,024, that is, every example data includes 1,024 points. We extracted 100 examples for each severity condition, and thus the D_MIX and D_ORF dataset consists of 400 examples, simultaneously, the D_IRF and D_BF datasets contain 500 examples, respectively. The detailed description with respect to the experimental datasets is presented in Table 2, where “07”, “14”, “21” and “28” mean that fault diameter is 0.007, 0.014, 0.021 and 0.028 inches. For verifying the proposed scheme in this study, the overall datasets are split into two portions, *i.e.*, training datasets (50%) and test datasets (50%).

**Figure 4.**
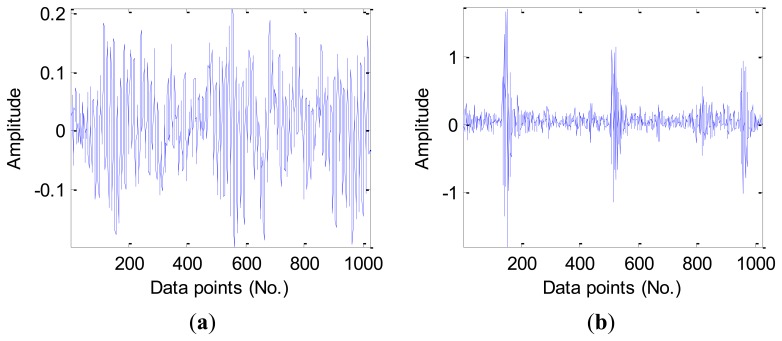

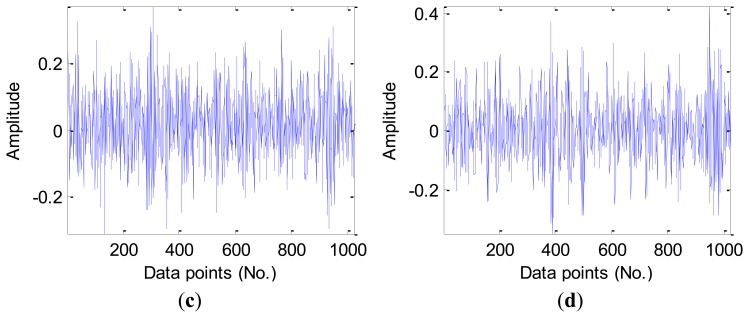
The vibration signal waveforms from the different fault types: (**a**) Health bearing. (**b**) Inner race fault. (**c**) Outer race fault. (**d**) Ball fault.

Figure 4 presents the vibration signal waveforms from four signal samples of the different fault types in the D_MIX dataset, note that there is a manifest difference in the overall vibration magnitude for the new health bearing when compared with other three types of fault bearings. Nevertheless, we still need to process the signal (calculate signal features) due to very high dimensions of the original vibration signals.

### 4.2. Signal Processing

**Figure 5.**
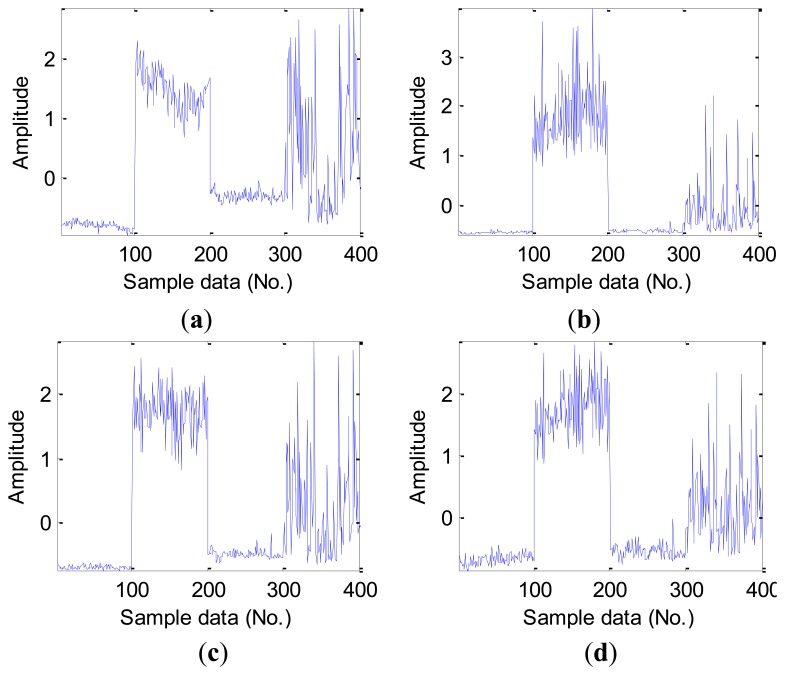
The normalized time domain features in the D_MIX dataset: (**a**) RMS. (**b**) kurtosis value. (**c**) peak-peak value. (**d**) impulse factor. (Note: Sample data No. 1–100, 101–200, 201–300,301–400 represent Normal, IRF14, ORF14 and BF14, respectively).

For the obtained vibration signal data, we calculate original features following the time domain, frequency domain and time-frequency domain for the next feature extraction. Time domain features could be calculated directly from vibration signals using Equations (1)–(10). For validating the employed time domain features in this work, Table 3 lists the average value of the statistical time domain features in the D_MIX dataset. It can be seen from Figure 5, there are some differences in the various fault types of bearings in the D_MIX dataset, but some existed differences is still not easy to be distinguished, especially in the ball fault bearings.

**Figure 6.**
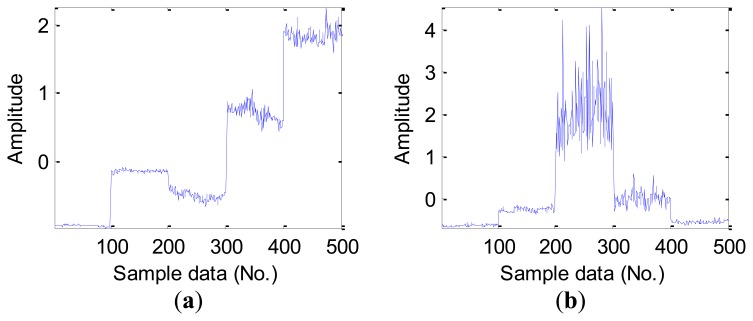

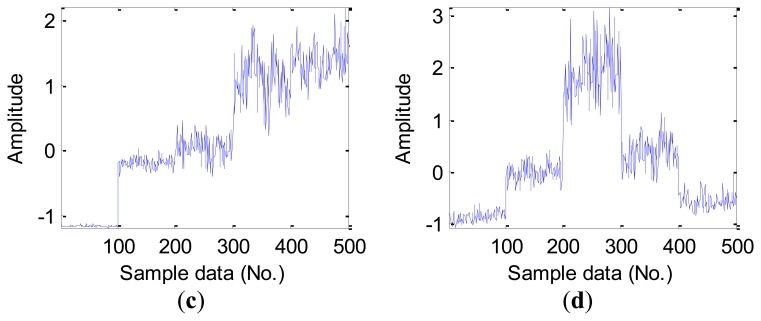
The normalized time domain features in the D_IRF dataset: (**a**) RMS. (**b**) kurtosis value. (**c**) peak-peak value. (**d**) impulse factor. (Note: Sample data No. 1–100, 101–200, 201–300, 301–400, 401–500 represent Normal, IRF07, IRF14, IRF21 and IRF28 respectively.)

As mentioned earlier, furthermore, some statistical features of time domain are sensitive to inchoate faults, for instance, RMS and kurtosis values should be able to capture the mutual difference in the time domain signal for the fault and healthy bearings. Figure 6 shows this character of four statistical features of time domain in the D_IRF dataset, we note that the feature of RMS can recognize differences in four bearing conditions, however, kurtosis values, peak-peak value and impulse factor only can capture better infancy fault, where it shows poor ability to identify much more severe faults.

**Figure 7.**
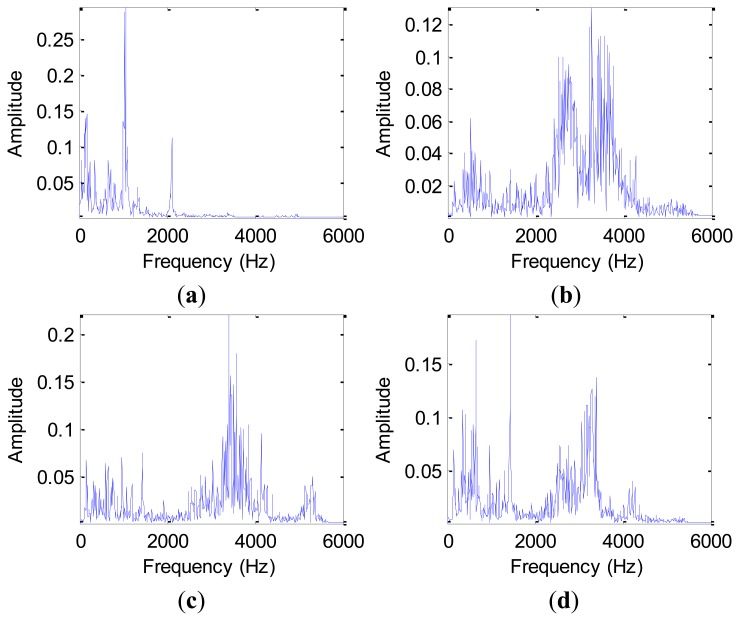
The single-sided amplitude spectrum based on FFT in the D_MIX dataset: (**a**) the normal bearing. (**b**) IRF14 bearing. (**c**) ORF14 bearing. (**d**) BF14 bearing.

**Figure 8.**
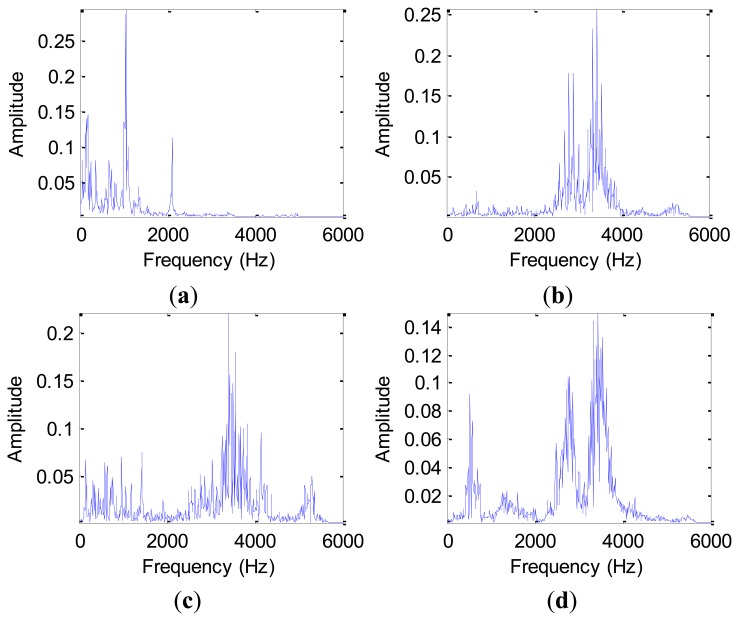
The single-sided amplitude spectrum based on FFT in the D_ORF dataset: (**a**) the normal bearing. (**b**) ORF07 bearing. (**c**) ORF14 bearing. (**d**) ORF21 bearing.

The advantage of frequency domain analysis over time domain analysis is its ability to easily identify and isolate certain frequency components of interest. The most widely used conventional analysis is the spectrum analysis by means of fast Fourier transform (FFT), which is a well-established method because of its simplicity. Figure 7 shows the spectrum based on FFT for a normal sample and three different fault samples in the D_MIX dataset, and Figure 8 displays the corresponding spectrum for a normal bearing and three outer race fault bearings with faulty diameter: 0.007 (ORF07), 0.014 (ORF14) and 0.021 (ORF21) inches in the D_ORF dataset, respectively.

The Fourier spectrum analysis provides a general method for examining the global energy-frequency distribution. The main idea of spectrum analysis is to either look at the whole spectrum or look closely at certain frequency components of interest and thus extract features from the obtained vibration signal data. On this basis, we calculate frequency domain features, such as frequency center, RMS frequency and root variance frequency using Equations (11)–(13). However, the features from the FFT analysis results tend to average out transient vibrations and don't provide a wholesome measure of bearing health states. Therefore, one manifest limitation of frequency domain analysis is its inability to handle non-stationary waveform signals, which are very common when machinery faults occur [33].

**Figure 9.**
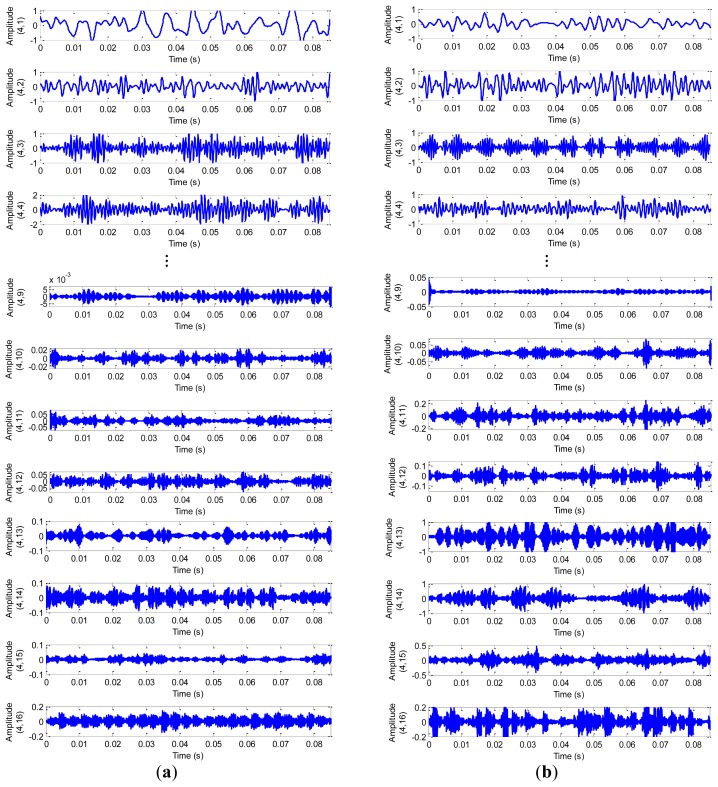
The signals of the decomposed by WPT from: (**a**) the normal bearing. (**b**) the ball fault bearing.

Time-frequency analysis, which investigates waveform signals in both time and frequency domain, has been developed for non-stationary waveform signals. Traditional time-frequency analysis uses time-frequency distributions, which represent the energy or power of waveform signals in two-dimensional functions of both time and frequency to better reveal fault patterns for more accurate diagnosis. In this study, we decompose vibration signals obtained from the test-rig into four scales using WPT with mother wavelet ‘DB4’, Figure 9 displays the original and decomposed signals from a normal bearing sample and a ball fault bearing sample in the D_BF dataset, therein, we list only eight decomposed signals for the purpose of simplifying indication.

**Figure 10.**
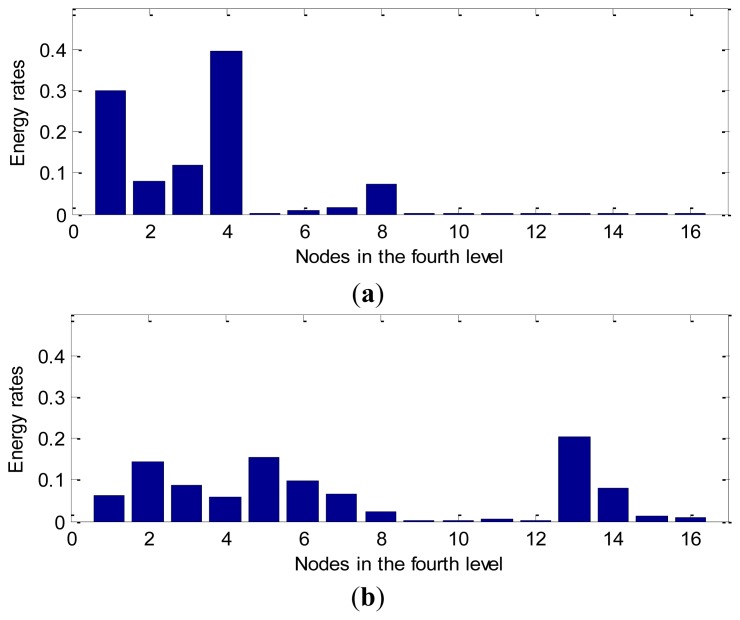
The normalized feature analysis of wavelet packet node energy in Figure 9: (**a**) the normal bearing. (**b**) the BF14 bearing.

From Figure 9, we note that there is a relatively large difference between the normal bearing and the ball fault bearing, especially at the high frequency of the decomposed signals. For the purpose of comparison, we calculate the average value of the wavelet packet nodes energy from decomposed signals of the normal bearings and the ball fault bearings with faulty diameter: 0.007 (BF07), 0.014 (BF14), 0.021 (BF21) and 0.028 (BF28) inches in the D_BF dataset using Equation (16), respectively. The normalized wavelet packet energy was analyzed from the corresponding sixteen decomposed signal nodes, the results are shown in Figure 10, its distribution of energy are different mutually.

### 4.3. Feature Extraction

In the technique presented in this paper, the total 29 features were calculated from 10 time domain features, three frequency domain features and 16 time-frequency domain features. In general, it is difficult to estimate which features are more sensitive to fault development and propagation in a machine system, furthermore, the effectiveness of these original features could change under different working conditions. In addition, this amount of original features is too many, thus it could be a burden and decrease the performance of the classifier or recognizer. Therefore, feature extraction and dimension reduction using some related technique are proposed in this study, so that more salient and low dimensional features are extracted for performing bearings fault diagnosis or prognosis.

**Figure 11.**
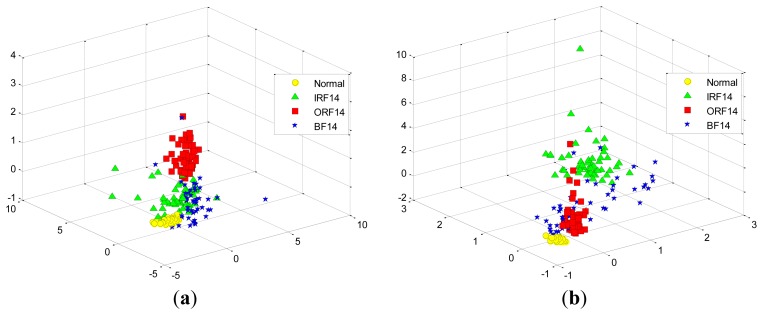
The randomly selected three features from the total 29 features in the D_MIX dataset: (**a**) the first selection. (**b**) the second selection.

**Figure 12.**
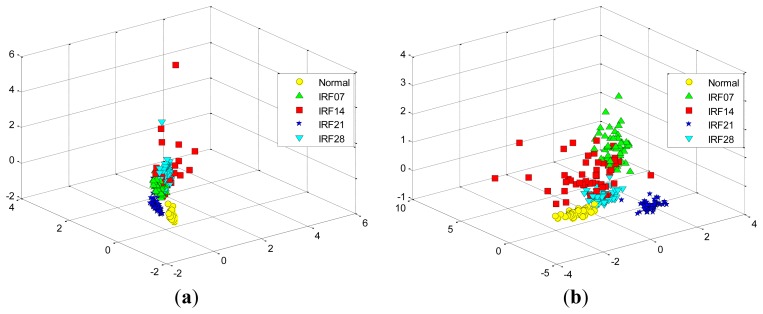
The randomly selected three features from the total 29 features in the D_IRF dataset: (**a**) the first selection. (**b**) the second selection.

At first, we take two experiments, each select randomly three features from the total 29 features in the D_MIX dataset, which are illustrated in Figure 11(a,b), respectively. Similarly, we also select randomly three features in the D_IRF dataset, the first and the second selected features are presented in Figure 12(a,b), respectively. It is shown that these features cannot separate well among the conditions of bearing fault because of high-dimensional data tends to redundancy, therefore, we cannot input them into the classifier directly.

In order to validate the performance of SR-based method for feature extraction, SR is originally implemented in the D_MIX dataset, the first *d* eigenvectors corresponding to the large *d* eigenvalues are selected to implement data projection using Equation (32).

**Figure 13.**
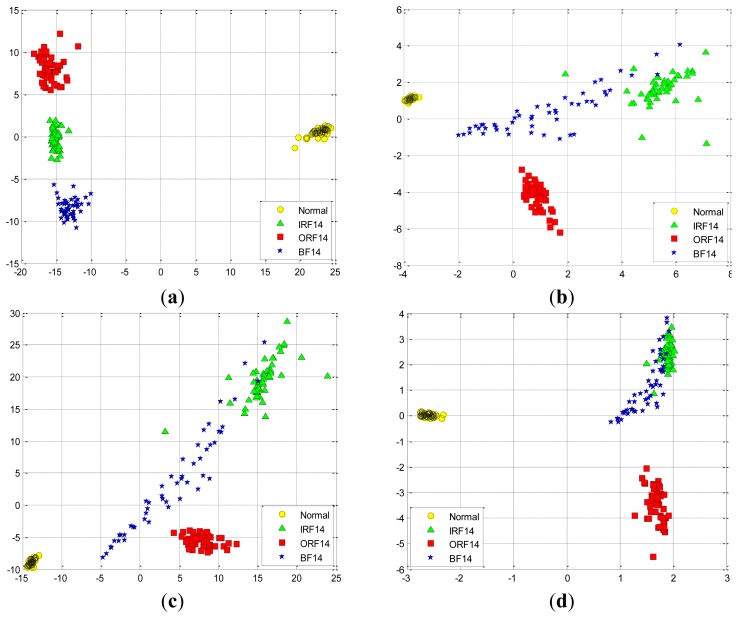
The data projection result with the first two eigenvectors in the D_MIX dataset using: (**a**) SR. (**b**) PCA. (**c**) FA. (**d**) LPP.

**Figure 14.**
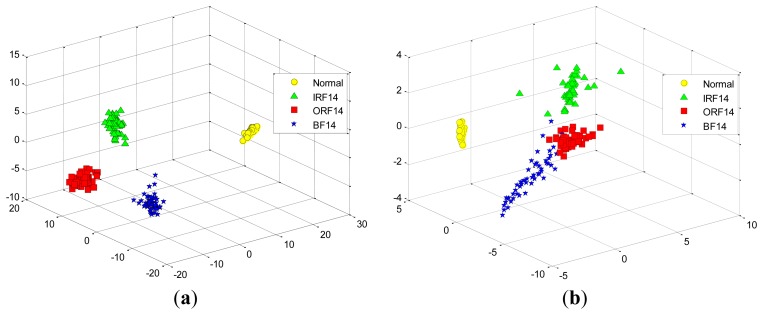

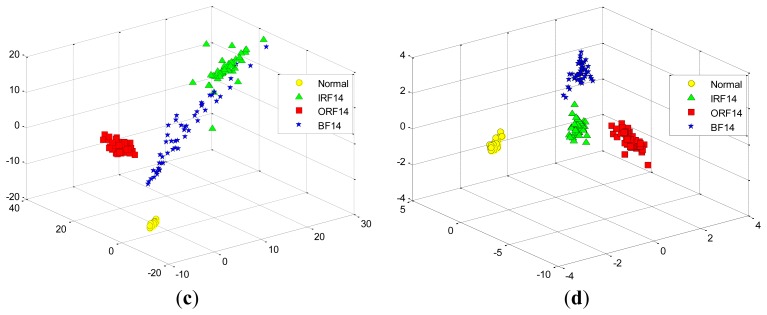
The data projection result with the first three eigenvectors in the D_MIX dataset using: (**a**) SR. (**b**) PCA. (**c**) FA. (**d**) LPP.

Figure 13(a) shows the data projection result with the first two eigenvectors corresponding to the large two eigenvalues, where the first two projected column vectors are plotted. For the purpose of the comparison, the projected results using PCA, FA and LPP are also illustrated in Figure 13(b–d), respectively. In addition, Figure 14 presents corresponding comparison of the data projection result with the first three eigenvectors.

We generally keep the first several eigenvectors corresponding to the large eigenvalues which can keep most variance information of the given data. However, high input data dimensions could decrease the recognition performance of the classifiers and result in more training time cost. Thus, the selection of the number of the eigenvectors should be based on the requirement of the real-world applications [1]. In this study, we select the first three eigenvectors of data projection result for inputting the classifiers, and we also display the first two eigenvectors of data projection result for visualization well. As shown in Figures 13 and 14, it is obvious that the data projection result with the first two or three eigenvectors using SR outperforms other methods in the D_MIX dataset.

**Figure 15.**
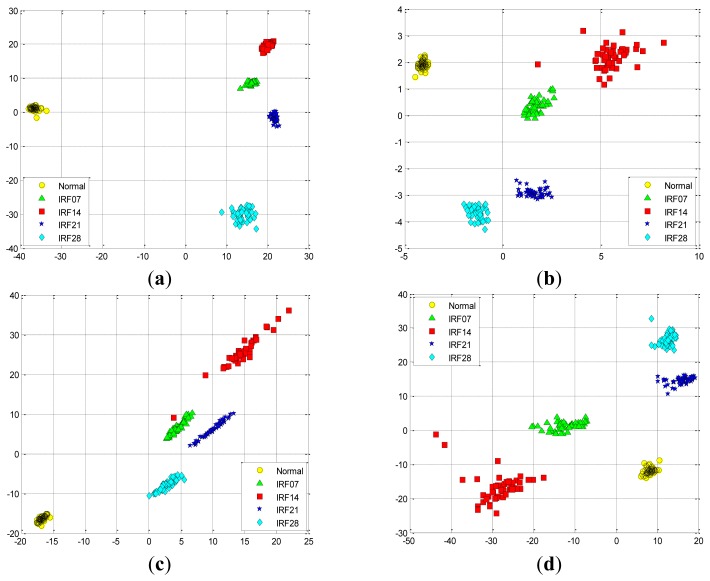
The data projection result with the first two eigenvectors in the D_IRF dataset using: (**a**) SR. (**b**) PCA. (**c**) FA. (**d**) LPP.

**Figure 16.**
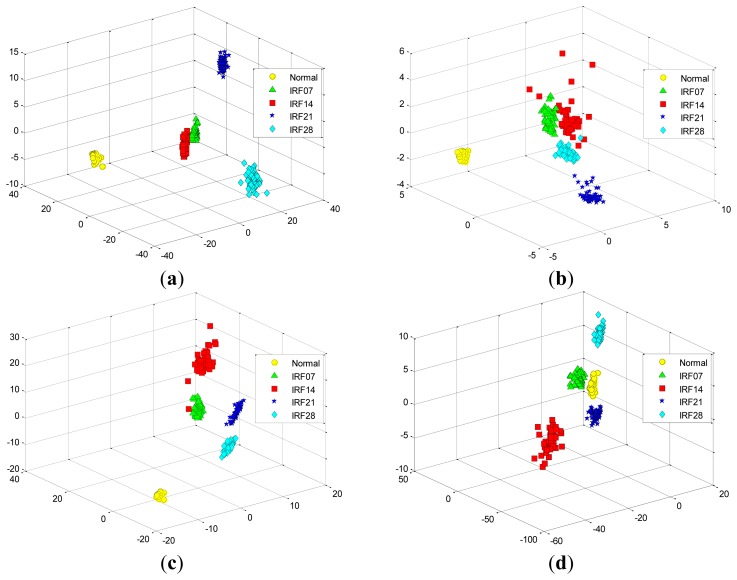
The data projection result with the first three eigenvectors in the D_IRF dataset using: (**a**) SR. (**b**) PCA. (**c**) FA. (**d**) LPP.

Similarly, we also perform these four feature extraction algorithms in the D_IRF dataset, the extracted first two and three features are compared in Figures 15 and 16, respectively. Severity recognition references to the identification of the differentiation of defective states of the bearings, e.g., normal, IRF07, IRF14, IRF21, IRF28 in the D_IRF dataset.

From the corresponding compared results, we can observe that SR has better projection performance over other three methods, as it can obtain a more clear separation of the clustering on the map for the corresponding severity recognition. This is due to the fact that SR is capable of discovering local structured information of the data manifold. However, PCA aims to discover the global structure of the Euclidean space. For the D_IRF dataset, each of fault severity classes is a local structure, SR preserves the intrinsic geometry structure of the dataset in a low-dimensional space. This illustrates that the local information could be more meaningful than the global information from given dataset in some industrial situations. In addition, LPP shows better performance than PCA and FA, since LPP is also graph embedding method based on the local structure of the manifold. This result indicates that features extracted via spectral graph embedding analysis could be more effective than which extracted via global structure by PCA and FA, which illustrates that SR-based feature extraction is very effective to extract most sensitive features for fault classification and severity recognition tasks. As we know, the clearer the separation, the more robust a classifier is. Consequently, the extracted features by SR are able to improve the performance of the classifiers more effectively, which further proves that SR is capable of extracting the most effective features from original features without too much calculation cost.

### 4.4. Method Evaluation

**Figure 17.**
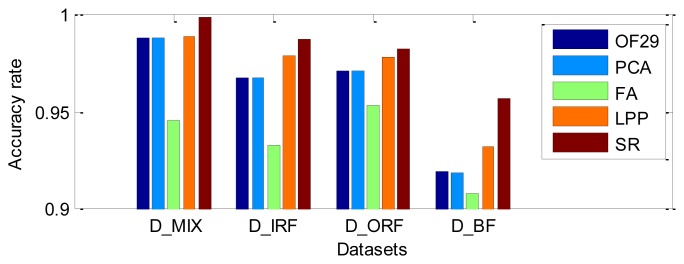
The comparison of the accuracy rate in four datasets.

In this study, *K*-Means is adopted to evaluate the performance of SR, PCA, FA and LPP. The first three extracted features corresponding to the largest eigenvalues are employed as the input features of *K*-Means. *K*-Means was implemented to recognize the clusters of the different bearing fault types, the acquired training dataset and testing dataset are used for modeling *K*-Means and checking misclassification, respectively. For given dataset, the accuracy rates are presented in Table 4, the classification results based on the original 29 features (OF29) and the first three features extracted by SR, PCA, FA and LPP are also presented in Table 4 and Figure 17. It can be observed that PCA and FA don't improve the recognition performance of *K*-Means in comparison with using OF29, both LPP and SR improve the accuracy rate, respectively. The results of this experiment are consistent with the actual situation of the CWRU dataset, since the data quality of the artificially introduced faults on bearings is very good, thus the features in different fault conditions are pretty separable. The *K*-Means recognized relatively accurately all of the different severity classes through the use of the methods based on PCA, LPP and SR. In addition, it is shown that the SR all gives more satisfied results as compared to others in four datasets, this further demonstrates the effectiveness of SR for feature extraction or dimensionality reduction of the given input space, and also be confirmed to improve the performance of the classifier obviously. Therefore, we can safely make use of SR in order to extract the most effective features among the practical applications.

In order to further evaluate the proposed SR-based method, we adopt other experimental data, in particular, the bearing fault data acquired from an accelerated bearing life tester (ABLT-1) at the Hangzhou Bearing Test and Research Center in China (detailed information is described in [1]). The differentiation of fault states of bearings include three classes: normal, slightly degradation, and severe degradation (failure). The fault conditions can be estimated by the magnitude of the representative features, which are produced by the effective feature extraction methods. For this case, we collect the data from the whole life of the bearing to implement fault classification, and randomly select 100 samples from each fault states, and thus 300 samples are collected for the test bearing, 50% of samples are used as the training set to construct *K*-means model, while the remaining 50% of samples are used as the testing set to test the classification accuracy rate of *K*-means using the first three extracted features corresponding to the largest eigenvalues.

In this case, we not only compare with PCA, FA and LPP, but also compare with some other Graph Embedding based approaches, such as Laplacian Eigenmap (LE) and Linear Discriminant Analysis (LDA). The experimental results of *K*-means is shown in Table 5, the accuracy rate of classification by *K*-Means using the features extracted by SR is significantly better than that of *K*-Means using the features extracted by other methods, SR shows a similar performance with the supervised-based LDA. In addition, we can observe that there are some differences in the computational time of feature extraction consumed among the several methods. In Table 5, the computational time of LDA method is the highest, although it seeks the projective functions which are very perfect in the training set and testing set, so it is computationally expensive. The PCA method fails to show more improvement in the computational time; this is probably due to the fact that PCA does not encode discriminating information. The SR method achieves significantly better performance than other methods, which suggests that SR only needs to solve *c*-1 regularized least squares problems which are very efficient, This nice property makes it possible to apply SR to high dimensional large datasets in real-world applications. We also note that the classification accuracy rate of LPP-based and LE-based methods are also relatively higher, this is mostly due to the fact that the structured information in the experimental data is very important for feature extraction. Fortunately, SR is similar to them, and it is capable to discover local structured information in the data manifold. Specifically, this important property may enable SR to find more meaningful low-dimensional information hidden in the high-dimensional features compared with PCA and FA methods. Overall, this case also further demonstrates that the SR-based feature extraction method is very effective to improve the performance of classifiers.

It is noted that we tested the performance of the SR processing using the whole training and testing data for feature extraction in this experiment, which is not related to new test samples. In fact, handling data out samples (*i.e.*, new inputs) problems presents a big challenge in the area of feature extraction. Due to space limitation, this problem is not discussed detail in the paper. In the real-world application, we should firstly transfer the training data into the project space under the weight matrix ***W***, then using the same weight matrix ***W*** to treat the new testing data.

## 5. Conclusions

This paper has proposed a novel fault feature extraction scheme by adopting SR for bearing accelerometer sensor signals, and is the first time SR was applied to feature extraction of bearing faults. SR combines the spectral graph analysis and regression to provide an efficient and effective approach for regularized subspace learning problems, so that it can extract the most representative features from original calculated features. We adopt *K*-Means to evaluate the performance of the proposed feature extraction approaches, and the experimental results on obtained bearing vibration signal data have revealed that SR yields higher classification rates than other similar approaches, such as PCA, FA and LPP *etc.* According to this result, we conclude that the SR-based feature extraction scheme has great potential to be an effective and efficient tool for bearing fault diagnosis and prognosis, and the application of the SR-based method can serve as a promising alternative for intelligent maintenance systems in the future.

## Figures and Tables

**Table 1.** The 29 initial features from vibration signals.

**Types**	**Features**
**Time domain**	RMS, SRA, KV, SV, PPV, CF, IF, MF, SF, KF
**Frequency domain**	FC, RMSF, RVF
**Time-frequency domain**	WPNE(4,1), WPNE(4,2), …, WPNE(4,16)

**Table 2.** The experimental datasets.

**Datasets**	**Number**	**Fault type and diameter**	**Description**
**D_IRF**	500	Normal, IRF07, IRF14, IRF21, IRF28	inner race fault severity
**D_ORF**	400	Normal, ORF07, ORF14, ORF21	outer race fault severity
**D_BF**	500	Normal, BF07, BF14, BF21, BF28	ball fault severity
**D_MIX**	400	Normal, IRF14, ORF14, BF14	mixed fault classification

**Table 3.** The average value of the time domain features in the D_MIX dataset.

**Features**	**Normal**	**IRF14**	**ORF14**	**BF14**
**RMS**	0.073 ± 0.003	0.194 ± 0.017	0.100 ± 0.004	0.141 ± 0.054
**SRA**	0.050 ± 0.003	0.0878 ± 0.007	0.068 ± 0.003	0.080 ± 0.023
**KV**	2.760 ± 0.192	22.252 ± 5.486	3.003 ± 0.237	6.509 ± 4.491
**SV**	−0.032 ± 0.098	−0.050 ± 0.187	−0.001 ± 0.066	0.052 ± 0.196
**PPV**	0.419 ± 0.031	3.028 ± 0.390	0.645 ± 0.069	1.408 ± 0.844
**CF**	3.038 ± 0.289	8.079 ± 0.937	3.411 ± 0.344	5.061 ± 1.291
**IF**	3.770 ± 0.389	13.603 ± 1.797	4.277 ± 0.448	7.134 ± 2.533
**MF**	4.429 ± 0.480	17.870 ± 2.389	5.051 ± 0.536	8.907 ± 3.571
**SF**	1.240 ± 0.015	1.682 ± 0.060	1.254 ± 0.012	1.382 ± 0.130
**KF**	2.755 ± 0.198	20.998 ± 5.237	3.000 ± 0.220	6.508 ± 4.492

**Table 4.** The accuracy rate of classification by *K*-Means in four datasets.

**Datasets**	**OF29**	**PCA**	**FA**	**LPP**	**SR**
**D_MIX**	0.9882	0.9881	0.9457	0.9893	**0.9987**
**D_IRF**	0.9678	0.9676	0.9331	0.9793	**0.9876**
**D_ORF**	0.9716	0.9715	0.9536	0.9782	**0.9829**
**D_BF**	0.9194	0.9191	0.9085	0.9324	**0.9571**

**Table 5.** The accuracy rate of classification by *K*-Means in the bearing fault data from ABLT-1.

**Methods**	**Computational time (s)**	**Training accuracy rate (%)**	**Testing accuracy rate (%)**
**SR**	**2.509**	**0.9012**	**0.8426**
**PCA**	4.296	0.8163	0.7231
**FA**	6.348	0.7615	0.6912
**LPP**	4.973	0.8651	0.7984
**LE**	4.397	0.8425	0.7661
**LDA**	7.242	0.8987	0.8214

## References

[1] Yu J. (2011). Bearing performance degradation assessment using locality preserving projections and Gaussian mixture models. Mech. Syst. Sign. Process..

[2] Wang Y., Kang S., Jiang Y., Yang G., Song L., Mikulovich V. (2011). Classification of fault location and the degree of performance degradation of a rolling bearing based on an improved hyper-sphere-structured multi-class support vector machine. Mech. Syst. Sign. Process..

[3] Yang Z., Cai L., Gao L., Wang H. (2012). Adaptive redundant lifting wavelet transform based on fitting for fault feature extraction of roller bearings. Sensors.

[4] Gao L., Yang Z., Cai L., Wang H., Chen P. (2011). Roller bearing fault diagnosis based on nonlinear redundant lifting wavelet packet analysis. Sensors.

[5] Siegel D., Al-Atat H., Shauche V., Liao L., Snyder J., Lee J. (2012). Novel method for rolling element bearing health assessment—A tachometer-less synchronously averaged envelope feature extraction technique. Mech. Syst. Sign. Process..

[6] van Wyk B.J., van Wyk M.A., Qi G. (2009). Difference histograms: A new tool for time series analysis applied to bearing fault diagnosis. Patt. Recog. Lett..

[7] Pan Y., Chen J., Li X. (2010). Bearing performance degradation assessment based on lifting wavelet packet decomposition and fuzzy c-means. Mech. Syst. Sign. Process..

[8] Widodo A., Yang B.S. (2007). Application of nonlinear feature extraction and support vector machines for fault diagnosis of induction motors. Expert Syst. Appl..

[9] Yang L., Lv J., Xiang Y. (2012). Underdetermined blind source separation by parallel factor analysis in time-frequency domain. Cognit. Comput..

[10] Liao L., Lee J. (2009). A novel method for machine performance degradation assessment based on fixed cycle features test. J. Sound Vibr..

[11] Widodo A., Yang B.S. (2011). Machine health prognostics using survival probability and support vector machine. Expert Syst. Appl..

[12] Côme E., Oukhellou L., Denœux T., Aknin P. (2012). Fault diagnosis of a railway device using semi-supervised independent factor analysis with mixing constraints. Patt. Anal. Appl..

[13] Yu J.B. (2011). Bearing performance degradation assessment using locality preserving projections. Expert Syst. Appl..

[14] Li B., Zhang P., Liu D., Mi S., Ren G., Tian H. (2011). Feature extraction for rolling element bearing fault diagnosis utilizing generalized S transform and two-dimensional non-negative matrix factorization. J. Sound Vibr..

[15] Cai D. (2009). Spectral regression: A regression framework for efficient regularized subspace learning. Ph.D. Thesis.

[16] Cai D., He X., Han J. (2011). Speed up kernel discriminant analysis. VLDB J..

[17] Wang C., Chen J., Sun Y. (2010). Sensor network localization using kernel spectral regression. Wirel. Commun. Mobile Comput..

[18] Lin G.F., Zhu H., Fan Y.D., Fan C.X. (2011). Human action recognition based on random spectral regression. Artif. Intell. Comput. Intell..

[19] Zhang B., Gao Y. (2012). Spectral regression dimension reduction for multiple features facial image retrieval. Int. J. Biometr..

[20] Garg G., Singh V., Gupta J.R.P., Mittal A. (2012). Wrapper based wavelet feature optimization for EEG signals. Biomed. Eng. Lett..

[21] Yaqub M., Gondal I., Kamruzzaman J. (2012). Inchoate fault detection framework: Adaptive selection of wavelet nodes and cumulant orders. IEEE Trans. Instrum. Measur..

[22] Wang H., Chen P. (2009). A feature extraction method based on information theory for fault diagnosis of reciprocating machinery. Sensors.

[23] Peng Z., Zhang W., Lang Z., Meng G., Chu F. (2011). Time–frequency data fusion technique with application to vibration signal analysis. Mech. Syst. Sign. Process..

[24] Han T., Yang B.S., Choi W.H., Kim J.S. (2006). Fault diagnosis system of induction motors based on neural network and genetic algorithm using stator current signals. Int. J. Rotat. Mach..

[25] Goumas S.K., Zervakis M.E., Stavrakakis G. (2002). Classification of washing machines vibration signals using discrete wavelet analysis for feature extraction. IEEE Trans. Instrum. Measur..

[26] Rafiee J., Tse P., Harifi A., Sadeghi M. (2009). A novel technique for selecting mother wavelet function using an intelli gent fault diagnosis system. Expert Syst. Appl..

[27] Yu J. (2012). Health condition monitoring of machines based on hidden markov model and contribution analysis. IEEE Trans. Instrum. Measur..

[28] Yu T., Yuan Z., Dai F. Spectral regression based subspace learning for face recognition.

[29] Cai D., He X., Han J. Spectral regression for efficient regularized subspace learning.

[30] Luu K., Dai Bui T., Suen C.Y., Ricanek K. Spectral regression based age determination.

[31] Li Z., Yan X., Jiang Y., Qin L., Wu J. (2012). A new data mining approach for gear crack level identification based on manifold learning. Mechanics.

[32] Loparo K. Bearings vibration data set, case western reserve university. http://www.eecs.case.edu/laboratory/bearing/welcome_overview.htm.

[33] Jardine A.K.S., Lin D., Banjevic D. (2006). A review on machinery diagnostics and prognostics implementing condition-based maintenance. Mech. Syst. Sign. Process..

